# Identification of Epstein-Barr Virus Replication Proteins in Burkitt’s Lymphoma Cells

**DOI:** 10.3390/pathogens4040739

**Published:** 2015-10-30

**Authors:** Chris Traylen, Sharada Ramasubramanyan, Jianmin Zuo, Martin Rowe, Rajaei Almohammad, Kate Heesom, Steve M. M. Sweet, David A. Matthews, Alison J. Sinclair

**Affiliations:** 1 School of Life Sciences, University of Sussex, Brighton BN1 9QG, UK; E-Mails: C.Traylen@sussex.ac.uk (C.T.); sramasubramanyan@gmail.com (S.R.); r.almohammed@sussex.ac.uk (R.A.); 2 School of Cancer Sciences and Centre for Human Virology, University of Birmingham College of Medical and Dental Sciences, Edgbaston, Birmingham B15 2TT, UK; E-Mails: j.zuo@bham.ac.uk (J.Z.); m.rowe@bham.ac.uk (M.R.); 3 Genome Damage and Stability Centre, University of Sussex, Brighton BN1 9RQ, UK; E-Mail: ss641@sussex.ac.uk; 4 University of Proteomics Facility, Biomedical Sciences Building, Bristol BS8 1TD, UK; E-Mail: K.Heesom@bristol.ac.uk (K.H.); 5 School of Cellular and Molecular Medicine, University of Bristol, Biomedical Sciences Building, Bristol BS8 1TD, UK; E-Mail: padam@bristol.ac.uk (D.A.M.)

**Keywords:** virus, cancer, replication, proteome, herpes, Epstein-Barr

## Abstract

The working model to describe the mechanisms used to replicate the cancer-associated virus Epstein-Barr virus (EBV) is partly derived from comparisons with other members of the Herpes virus family. Many genes within the EBV genome are homologous across the herpes virus family. Published transcriptome data for the EBV genome during its lytic replication cycle show extensive transcription, but the identification of the proteins is limited. We have taken a global proteomics approach to identify viral proteins that are expressed during the EBV lytic replication cycle. We combined an enrichment method to isolate cells undergoing EBV lytic replication with SILAC-labeling coupled to mass-spectrometry and identified viral and host proteins expressed during the EBV lytic replication cycle. Amongst the most frequently identified viral proteins are two components of the DNA replication machinery, the single strand DNA binding protein BALF2, DNA polymerase accessory protein BMRF1 and both subunits of the viral ribonucleoside-diphosphate reductase enzyme (BORF2 and BaRF1). An additional 42 EBV lytic cycle proteins were also detected. This provides proteomic identification for many EBV lytic replication cycle proteins and also identifies post-translational modifications.

## 1. Introduction

Epstein-Barr virus (EBV) is associated with diverse cancers including Burkitt’s lymphoma, Hodgkin’s lymphoma, NK/T lymphomas, Nasopharyngeal carcinoma and gastric cancer [1,2,3,4,5,6,7,8,9]. During the ~50-years since the identification of the virus [10] and the ~30 years since the genome sequence of the first isolate was published [11], there has been a strong focus on research into the viral genes commonly expressed in tumors, which has enabled us to obtain a good understanding of the ability of EBV to transform cells and so establish viral latency.

EBV within tumor cells undergoes lytic cycle replication only rarely and ~90% of EBV genes are not commonly expressed in tumors. However, these are transcribed following the disruption of latency as cells enter the EBV lytic replication cycle. Sensitive transcriptome analysis in Burkitt’s lymphoma cells that have been stimulated to initiate the EBV lytic replication cycle [12,13], together with array-based strategies [14,15] and earlier mapping approaches (reviewed in [16]), suggests that the entire genome complement is expressed once EBV lytic replication cycle is activated.

The contribution of several EBV lytic cycle genes has been subject to genetic evaluation. This identified BZLF1, BRLF1 [17], BSLF2 + BMLF1 [18] and BMRF1 [19] as essential for regulating viral gene expression during viral lytic replication and others (BFLF1, BFLF2, BFRF1, BGRF1 and BDRF1) contribute to encapsulating the viral genome [20,21,22]. In contrast, BGLF4 contributes to the efficiency of viral replication [23,24,25,26] and some viral genes such as BLLF1 and BNRF1 are not required to generate virus but rather contribute to the subsequent infection of cells or allow efficient entry and genome release [27,28,29]. Finally, some viral genes contribute to immune evasion of infected cells, e.g., BNLF2a [30]. The contributions that many other EBV lytic replication cycle genes make to the EBV lytic replication cycle are inferred through their homology with the alpha herpesvirus family [1]. Several of these proteins have been detected by immunofluorescence during viral replication (e.g., [31]).

Despite three studies using proteomics approaches [32,33,34], not all EBV lytic cycle genes have been previously identified and many have not been independently verified. Here, we used an engineered Burkitt’s lymphoma cell system to enrich for cells undergoing EBV lytic replication and coupled this with SILAC-proteomics to develop a route to detect EBV proteins in Akata cells undergoing EBV lytic replication. This allowed us to identify a total of 44 EBV proteins and post-translational modifications of several viral proteins.

## 2. Results

### 2.1. Isolation of Proteins in Cells Undergoing EBV Lytic Cycle

**Figure 1:**
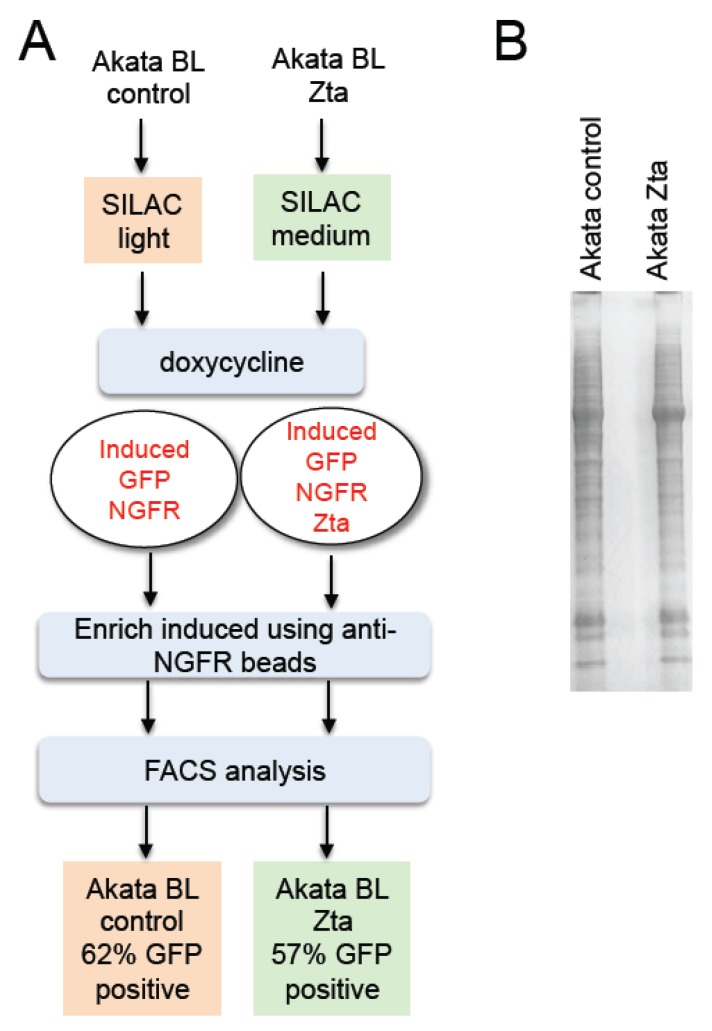
Enrichment of Burkitt’s Lymphoma (BL) cells induced to enter Epstein-Barr virus (EBV) lytic replication cycle. (**a**) Co-induction of Green Fluorescent Protein (GFP), Nerve Growth factor receptor (NGFR) and Zta (or not for control cells) and procedure to induce and enrich cells, together with the % enrichment (GFP positivity) is shown; (**b**) Total protein extracts were prepared, fractionated on SDS-PAGE and stained.

Cells from a Burkitt’s lymphoma which harbor EBV in type I latency had previously been engineered to co-express Green Fluorescent Protein (GFP), Nerve Growth Factor receptor (NGFR) and Zta (BZLF1) from an inducible bi-directional promoter (Akata-Zta). A cell line in which the Zta coding sequence is orientated in the non-coding direction acts as a control [35,36]. Proteins within the Akata control and Akata Zta cells were differentially metabolically labeled with amino acids consisting of stable isotopes. Following activation of the expression cassette using doxycycline, cells that had successfully been induced were isolated by their affinity for anti-NGFR coated magnetic beads. Analysis of GFP expression in the enriched cell population revealed a purity of between 57% and 62% (Figure 1).

### 2.2. Identification of Proteins in Cells Undergoing EBV Lytic Cycle

**Figure 2:**
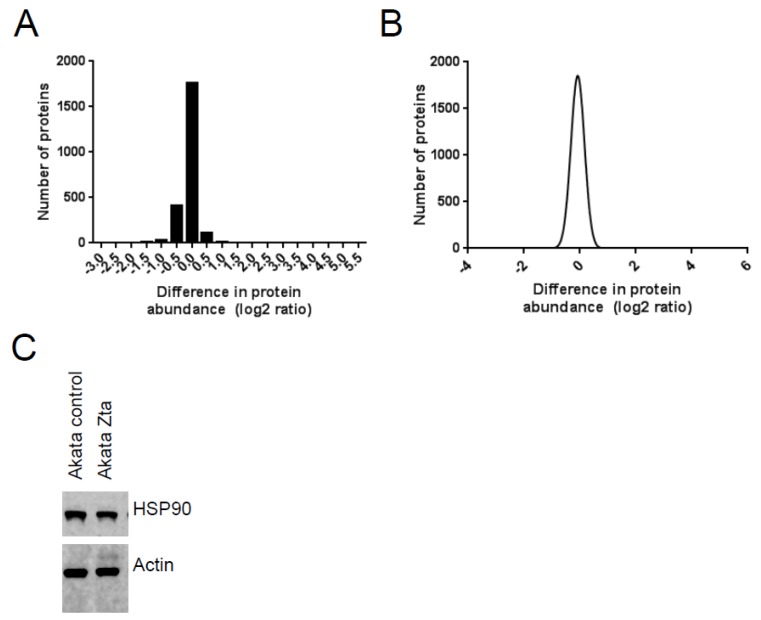
Stable isotope labeling with amino acids in cell culture (SILAC) coupled to mass spectrometry (MS) analysis of proteins in Burkitt’s Lymphoma (BL) cells during EBV lytic cycle. Total protein extracts were prepared from the enriched BL cells. MS analysis was undertaken. (**a**) The change in abundance of proteins with SILAC-information from both control and Zta expressing cells is shown; (**b**) The frequency distribution of the difference in protein abundance is shown as a Gaussian plot; (**c**) Total proteins were separated by SDS-PAGE. Western blots were probed with anti-HSP90 and beta actin antibodies.

The proteins from Akata-control and Akata-Zta were mixed in equal amounts and the relative abundance of cellular and viral proteins was analyzed by quantitative mass spectrometry (MS). Global analysis of the differences in abundance detected through the differential SILAC labeling and the difference in abundance of individual proteins determined by Western blot analysis revealed a modest overall reduction in the abundance of cellular proteins (between 1.2 and 2-fold) during EBV lytic cycle (Figure 2). Viral proteins were identified only in the Zta-expressing cells.

### 2.3. Identification of EBV Proteins

To identify EBV proteins in cells undergoing lytic replication, we considered the peptides that match with an EBV protein. The Uniprot databases which include proteins from three viral genomes HHV4 (B95-8 UP000007640; AG876 UP000007639; and GD1 UP000007641). The identity of each of the 169 peptides that correspond to an EBV protein with a Posterior Error Probability (PEP) score of less than 1.0 × 10^−3^ are provided in Table S1. These were all up regulated ≥8.6 fold during lytic cycle, with the majority being undetectable in latency. This identified peptides corresponding to 33 EBV proteins (Table 1). In addition, a custom made database of the Akata EBV proteome was generated and searched to ensure that polymorphic regions were not overlooked. However, this revealed no additional protein identifications. In order to increase the sensitivity of EBV protein detection in our dataset, we carried out a further MaxQuant search against the EBV proteome (UniProt), omitting human sequences [37]. This identified an additional 11 EBV proteins, also shown in Table 1 (highlighted). The peptides associated with this search are listed in Table S2.

**Table 1:** EBV proteins identified by MS analysis.

Gene	Function
BALF2	Major DNA-binding protein
BALF4	Envelope glycoprotein B
BALF5	DNA polymerase catalytic subunit
BaRF1	Ribonucleoside-diphosphate reductase small chain
BBLF2-BBLF3	primase protein
BBLF4	DNA replication helicase
BBRF2	Virion egress protein UL7 homolog
BcLF1	Major capsid protein
BDLF1	Triplex capsid protein VP23 homolog
BFLF1	Packaging protein UL32 homolog
BFLF2	Virion egress protein
BFRF1	Virion egress protein UL34 homolog
BFRF3	Capsid protein VP26
BGLF2	Capsid-binding protein
BGLF4	Serine/threonine-protein kinase
BGLF5	Shutoff alkaline exonuclease
BHRF1	Apoptosis regulator
BKRF3	Uracil-DNA glycosylase
BLLF3	Deoxyuridine 5′-triphosphate nucleotidohydrolase
BLRF2	Tegument protein
BSLF2-BMLF1	mRNA export factor ICP27 homolog
BMRF1	DNA polymerase processivity factor
BNRF1	Major tegument protein
BORF2	Ribonucleoside-diphosphate reductase large subunit
BPLF1	Deneddylase
BRRF1	Transcriptional activator
BRRF2	Tegument protein
BSRF1	Tegument protein UL51 homolog
BTRF1	Uncharacterized protein BTRF1
BVRF2	Capsid scaffolding protein
BdRF1	
BXLF1	Thymidine kinase
BZLF1 *	Trans-activator protein
BDLF3	pg85
BLLF1	gp350
BMRF2	Protein BMRF2
BORF1	Triplex capsid protein
BPLF1	deneddylase
BRLF1	Replication and transcription factor
BRRF2	tegument protein
BSLF1	DNA primase
gH	gH
gL	gL
LF1	LF1

* BZLF1 expression is driven by the doxycycline induced expression vector in these cells so detection cannot be ascribed to the endogenous protein. Yellow highlight represents proteins only identified in the EBV-specific search.

**Table 2:** Post-translational modifications of EBV proteins identified by MS analysis.

Gene Name	Modification	pep_seq	aa of EBV Protein	Residue of Modification
BALF5	N terminal acetylation	[ac]SGGLFYNPFLRPNK	2–15	2
BLLF3	N terminal acetylation	[ac]MEACPHIR	9–16	9
BLRF2	Phosphorylation	GQPS[ph]PGEGTRPR	124–135	127
BMRF1	2 Phosphorylation	HTVS[ph]PSPS[ph]PPPPPR	330–343	333 and 337
BMRF2	N terminal acetylation	[ac]METTQTLR	1–8	1
BORF1	Phosphorylation	RLNIS[ph]R	26–31	30
BORF2	N terminal acetylation	[ac]ATTSHVEHELLSK	2–14	2
BXLF1	Phosphorylation	TQAAVTSNTGNS[ph]PGSR	86–101	97
BZLF1	N terminal acetylation	[ac]MMDPNSTSEDVK	1–12	1

**Figure 3:**
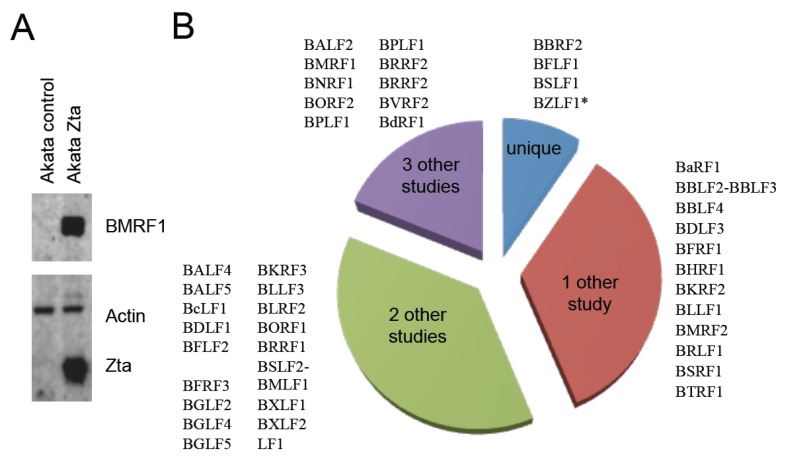
SILAC MS analysis of proteins EBV proteins detected in Akata cells during lytic cycle. (**A**) Akata control and Akata Zta cells were induced with doxycycline for 24 h and total protein extracts prepared. Western blot analysis of BMRF1, Zta and beta actin abundance is show; (**B**) The EBV proteins identified are shown in relation to previously published studies. BZLF1 is marked*, its expression is driven by the doxycycline induced expression vector in these cells so detection cannot be ascribed to the endogenous protein.

None of the EBV proteins are associated with EBV latency; all originate from genes with a characteristic lytic replication cycle pattern of expression [16]. One of the 44 proteins identified, BZLF1, could be derived from either the expression vector or the endogenous virus so it should not be considered as proof of identity of the endogenous protein. We confirm expression of one of these proteins, BMRF1, by Western blot (Figure 3A) and we show which gene products are uniquely identified here and which provides confirmation of proteins previously identified in other reports (Figure 3B).

### 2.4. Identification of Post-Translational Modifications of EBV Proteins

We searched for potential post-translational modifications of EBV proteins using MASCOT to search a minimal EBV database. All identifications were required to be from medium-labeled peptides, *i.e.*, present after EBV induction. The same phosphorylation and N-terminal acetylation modifications were also identified in a MaxQuant search of the EBV database. Those phospho-serine and amino-terminal acetylation modifications corresponding to proteins identified in Table 1 are shown in Table 2, with peptide identification evidence provided in Table S3. This identified that five lytic EBV proteins sustain amino-terminal acetylation; BZLF1 (Zta), BMRF2, BLLF3, BALF5, and BORF2. In addition EBV peptides corresponding to serine phosphorylation were identified for BMRF1, BLRF2, BORF1 and BXLF1.

## 3. Experimental Section

### 3.1. Cell Culture

Akata-Zta and Akata control cells [36] were cultured in RPMI–SILAC labeled RPMI containing 13C6-arginine and 4,4,5,5-D4-lysine (R6K4) (medium) and RPMI R0K0 (light) respectively (Dundee Cell products). Each was supplemented with 15% (v/v) dialyzed FBS and 100 units/mL penicillin, 100 μg/mL streptomycin and 2 mM L-glutamine (Life Technologies) at 37 °C with 5% CO_2_. Cells were maintained between 3 and 10 × 10^5^ cells/mL and were cultured in SILAC-medium for 16 population doublings. Doxycycline (Sigma) was added to a final concentration of 500 ng/mL and cells incubated for a further 24 h. Successfully induced cells were enriched using anti-NGFR antibodies coupled to paramagnetic beads as described [36].

### 3.2. FACS Analysis

Live cells were analyzed using a multi-parameter fluorescent activated cell analysis (FACs) (Facs Canto-Beckton Dickinson). GFP positive cells were identified using BD FACSDiva™ Software (Beckton Dickinson).

### 3.3. Western Blot Analysis

An equivalent number of cells were lysed using SDS-PAGE sample buffer at a final concentration of 1.0 × 10^4^ cells/μL. Extracts from 1.0 × 10^5^ cells were fractionated on SDS-PAGE. The total protein complement was detected following staining with Simply Blue stain or transferred to nitrocellulose. Proteins were identified using the following primary antibodies, beta actin (A2066, SIGMA), HSP90 (AC88 ab13492, ABCAM), Zta [38] and BMRF1 (8F92, ab30541, ABCAM). This was followed by incubation with species-specific infra-red labeled secondary antibodies (LiCor). The presence and relative abundance of proteins was determined using an Odyssey Fc Imager and Odyssey Image Studio (Licor).

### 3.4. Mass Spectrometry Collection and Analysis

Extracts from Zta expressing and not expressing cells were mixed and fractionated on SDS-PAGE (Novex). The lane was cut into six slices and each slice was subjected to in-gel digestion with a DigestPro MSi automatic digestion system (Intavis Bioanalytical Instruments) as described in [39]. The resulting peptides were fractionated using a Dionex Ultimate 3000 nano HPLC system coupled to an LTQ-Orbitrap Velos mass spectrometer (Thermo Scientific). In brief, peptides in 1% (v/v) formic acid were injected onto an Acclaim PepMap C18 nano-trap column (Dionex). After washing with 0.5% (v/v) acetonitrile 0.1% (v/v) formic acid, peptides were resolved on a 250 mm × 75 μm Acclaim PepMap C18 reverse phase analytical column (Dionex) over a 150 min organic gradient, using 7 gradient segments (1%–6% solvent B over 1 min, 6%–15% B over 58 min, 15%–32% B over 58 min, 32%–40% B over 3 min, 40%–90% B over 1 min, held at 90% B for 6 min and then reduced to 1% B over 1 min) with a flow rate of 300 nL∙min^−1^. Solvent A was 0.1% formic acid and Solvent B was aqueous 80% acetonitrile in 0.1% formic acid. Peptides were ionized by nano-electrospray ionization at 2.3 kV using a stainless steel emitter with an internal diameter of 30 μm (Thermo Scientific) and a capillary temperature of 250 °C. Tandem mass spectra were acquired using an LTQ-Orbitrap Velos mass spectrometer controlled by Xcalibur v2.1 software [40] and operated in data-dependent acquisition mode. The Orbitrap was set to analyze the survey scans at 60,000 resolution (at m/z 400) in the mass range m/z 300–2000 and the top six multiply charged ions in each duty cycle selected for MS/MS in the LTQ linear ion trap. Charge state filtering, where unassigned precursor ions were not selected for fragmentation, and dynamic exclusion (repeat count, 1; repeat duration, 30 s; exclusion list size, 500) were used. Fragmentation conditions in the LTQ were as follows: normalized collision energy, 40%; activation q, 0.25; activation time 10 ms; and minimum ion selection intensity, 500 counts. Data were acquired using the Xcalibar v2.1 software. The raw data files were processed and quantified using MaxQuant as described in [39] and searched against standard human proteome and EBV protein lists from UNIPROT and a translation of the Akata EBV genome. A search was also carried out against the EBV UniProt proteins plus contaminants, with the human sequences omitted [37]. Peptide precursor mass tolerance was set at 10 ppm, and MS/MS tolerance was set at 0.8 Da. Search criteria included carbamidomethylation of cysteine (+57.0214) as a fixed modification and oxidation of methionine (+15.9949) and appropriate SILAC labels as variable modifications.

Searches were performed with full tryptic digestion and a maximum of two missed cleavages was allowed. The reverse database search option was enabled and all peptide data was filtered to satisfy false discovery rate (FDR) of 1%.

A database search using Mascot was carried out against a database containing 282 EBV protein sequences from UniProt. The search used the following parameters: 10 ppm precursor mass tolerance; 0.6 Da fragment ion mass tolerance; fixed modification: carbamidomethylation (C); variable modifications: Protein N-terminus acetylation, methionine oxidation, phosphorylation (STY), 2H(4) K, 13C(6) R.

The mass spectrometry proteomics data have been deposited to the ProteomeXchange Consortium [1] via the PRIDE partner repository with the dataset identifier PXD002461 [41].

## 4. Conclusions

Two previous studies compared the proteomes in BL and primary effusion lymphoma (PEL) cells during EBV lytic cycle with the proteomes of cells during latency or to those that are refractory to entering EBV lytic cycle [33,34]. The previous studies used the histone deacetylase inhibitor sodium butyrate [34] and/or a combination of the histone deacetylase inhibitor sodium butyrate and 12-O-tetradecanoylphorbol-13-acetate to induce EBV to enter its lytic replication cycle. Here, we used a different method to initiate EBV lytic cycle gene expression, the ectopic expression of Zta protein. We previously demonstrated that this is sufficient to promote expression of several EBV lytic cycle genes leading to replication of the EBV genome [36]. Sensitive transcriptome analysis of EBV identified highly abundant mRNAs [12,14,33]. While some of the proteins encoded by these are readily detected in the lytic cells (e.g., BMRF1, BMLF1 and BHRF1), others are not detected in any study (e.g., BALF1). This highlights one of the limitations of interpreting a global proteomics study; some proteins do not generate peptides that can be unambiguously identified. Whether EBV completes the lytic cycle in response to any of these stimuli yet protein expression is too low to be detected by mass spectrometry, or whether the lytic cycles are aborted prior to full viral gene expression and release of infectious virus is unknown. In addition to the analysis of viral proteins within cells, proteins present in purified EBV virions have also been detected using proteomics [32].

A comparison of our data with these three datasets revealed that we detected 28 EBV proteins that had been identified in two or more previous studies. We therefore provide further support for the identification of these proteins. Importantly, our analysis detected 12 viral proteins that were only identified in one previous study, providing important independent evidence of their detection. In addition, we provide evidence for the first detection of three viral proteins by mass spectrometry. The first is BBRF2, which is a homologue of the HSV1 virion egress protein UL7. Clues as to its function arise from the recent demonstration that UL7 plays a role in linking tegument proteins of HSV1 to membranes [42]. The second protein is BFLF1. Interestingly, BFLF1 is a homologue of the HSV1 UL32 gene, which plays a role in HSV1 encapsidation [43] which supports the potential involvement of BFLF1 protein in cleavage and packaging of the viral genome [21]. The third, BSLF1, encodes the DNA primase that is required for genome lytic replication [44,45]. In addition, we detected Zta protein (BZLF1), although it is not possible to distinguish whether this originates from the endogenous genome or the expression vector.

Further analysis of the data identified evidence for novel post-translational modifications of nine EBV proteins. Amino-terminal acetylation events were identified for Zta, BLLF3, BALF5, BMRF2 and BORF2. For BALF5 and BORF2 amino terminal processing had occurred and the acetylation is present on the second residue, for the remainder it is present on the initiator methionine. Neither the acetylation nor the amino terminal processing had been described previously. A large sub-set of cellular proteins also sustain the amino terminal acetylation, the function is enigmatic, and roles in protein–protein interaction, sub-cellular targeting and degradation have all been postulated [46]. In addition to this, EBV peptides corresponding to serine phosphorylation of BMRF1, BLRF2, BORF1 and BXLF1 were identified. Of these, BMRF1 is known to be phosphorylated at residue 337 [47], in addition to residues 344, 349 and 355. We provide evidence for a further site of phosphorylation at serine 333. In addition, this is the first report that BLRF2, BORF2 and BXLF1 sustain serine phosphorylation.

In summary, the definitive identification of 44 EBV proteins in BL cells undergoing EBV replication and the identification of novel post-translational modifications of nine of these lytic cycle proteins increase the knowledge base of EBV lytic replication and may highlight different targets for future strategies to enable the development of therapeutic interventions to manipulate EBV replication.

## Supplementary Materials

Supplementary materials can be accessed at: http://www.mdpi.com/2076-0817/4/3/739/s1.
